# Comparative antilipidemic effect of N-acetylcysteine and sesame oil administration in diet-induced hypercholesterolemic mice

**DOI:** 10.1186/1476-511X-9-23

**Published:** 2010-03-06

**Authors:** Laskarina-Maria Korou, George Agrogiannis, Alkisti Pantopoulou, Ioannis S Vlachos, Dimitrios Iliopoulos, Theodoros Karatzas, Despoina N Perrea

**Affiliations:** 1 Department of Experimental Surgery and Surgical Research 'NS Christeas', Athens School of Medicine, Athens, Greece; 2 Department of Pathology, Athens School of Medicine, Athens, Greece

## Abstract

**Background:**

There is an increasing number of novel antilipidemic therapies under consideration. The putative hypolipidemic effect of N-acetylcysteine (NAC) and sesame oil was studied in a mouse model of dietary-induced hypercholesterolemia.

**Methods:**

Male C57bl/6 mice were assigned to the following groups: (NC) control group, (HC) group receiving test diet supplemented with 2% cholesterol and 0.5% cholic acid for 8 weeks, (HCN) group receiving the test diet with NAC supplementation (230 mg/kg p.o.) and (HCS) group fed the test diet enriched with 10% sesame oil. Total serum cholesterol, LDL-cholesterol, HDL-cholesterol and triglycerides were assayed at the beginning and at the end of the experiment. Total peroxides and nitric oxide (NO) levels were measured in the serum at the end of the experiment. Hepatic and aortic lesions were evaluated by haematoxylin-eosin staining.

**Results:**

Higher serum levels of total and LDL-cholesterol were recorded in all groups fed the high cholesterol diet. The HCN group presented reduced lipid levels compared to HC and HCS groups. No differences were observed between HCS and HC groups. Peroxide content in serum was markedly increased in mice consuming high cholesterol diet. NAC and sesame oil administration led to a significant decrease of serum lipid peroxidation in the levels of control group, whereas only NAC restored NO bioavailability. In terms of liver histology, the lesions observed in HCN group were less severe than those seen in the other high cholesterol groups.

**Conclusion:**

Co-administration of NAC, but not sesame oil, restored the disturbed lipid profile and improved hepatic steatosis in the studied diet-induced hypercholesterolemic mice. Both agents appear to ameliorate serum antioxidant defense.

## Background

Hypercholesterolemia in combination with raised LDL-cholesterol concentration represent major risk factors for the development and progression of atherosclerosis and consequently of cardiovascular disease [1]. Based on this evidence, scientific research is targeting the discovery of new drugs with hypocholesterolemic effects. Plant based dietary therapies and natural food components are being proposed nowadays for the prevention of dyslipidemia. The antioxidant N-acetylcysteine (NAC), an organosulfur from *Allium *plants and sesame oil appear as promising lipid-lowering agents in recent literature.

NAC is a hydrophilic cysteine-containing compound naturally formed in *Allium *plants such as garlic and onion. The intake of this compound was found to effectively decrease high saturated fat-induced triacylglycerol (TAG) and cholesterol accumulation in mice livers. Moreover it has been shown to protect the liver against high fat-induced oxidative damage [2].

Sesame belongs to the species *Sesamum indicum *(*Pedaliaceae *family). It has been extensively used as a traditional food in the orient but it is also considered to have important medical properties. There is abundant data available from different studies mainly on rodent species supporting the hypolipidemic action of many components of sesame seed and its oil [3,4]. Satchithanandam et al [4] concluded that the absorption of lymphatic cholesterol in rats fed diets containing sesame oil was about 50% less than by rats fed a control diet. Additionally, administration of non defatted sesame seed powder in hypercholesterolemic albino rats, improved their antioxidant status and significantly decreased plasma lipids [5]. To our knowledge there is no literature-based evidence of the potential hypolipidemic effects of sesame oil administration without substitution of other fat dietary compounds.

Therefore, the present study was undertaken to determine whether NAC and sesame oil administration would have beneficial hypolipidemic and antioxidant effects in a murine model consuming a chow diet, enriched with cholesterol and cholic acid.

## Methods

### Animals

12-week-old C57bl/6 male mice (*Mus musculus*) were obtained from Alexander Fleming Institute (Vari, Greece) and were acclimatised for one week before the experiment started. The animals were housed under conditions of controlled temperature (23 ± 2°C) and humidity (60%) with 12 h light/dark cycle. The experimental protocol was reviewed and approved by the Veterinary Directorate of Athens Prefecture and by the Ethics Committee of the Medicine School of the National and Kapodistrian University of Athens, in accordance with ethical recommendation of the European Communities Council Directive of November 24, 1986 (86/609/EEC).

### Experimental design

A total of 25 mice were divided into the following 4 experimental groups:

**NC **(n = 5): normal control with basal diet (4RF25, Mucedola, Milan, Italy); **HC **(n = 5): animals that received the normal chow diet supplemented with cholesterol (2%) and cholic acid (0.5%) (high cholesterol diet) for 8 weeks; **HCN **(n = 7): animals fed the high cholesterol diet with NAC (Flumil Antidoto, IΦET, Greece). NAC was administrated p.o. in drinking water in a concentration tested to result in a daily uptake of approximately 230 mg/kg body weight. The dosage of the drug was determined according to previous reports on rodents [6,7]. **HCS **(n = 8): mice which received the high cholesterol diet enriched with 10% sesame oil for the same period. Sesame oil was obtained from a local health food store. High cholesterol content was used in the high cholesterol diet in order to obtain hypercholesterolemia after a short follow-up period. Cholesterol and cholic acid (approximately 95% and 99% purity respectively) were obtained from Sigma-Aldrich (Germany). The high cholesterol diet was prepared weekly by dissolving cholesterol in diethyl ether (without butylated hydroxytoluene as inhibitor) and cholic acid in methanol and thoroughly coating with these solutions the pellets of standard chow cut in small pieces. After solvent evaporation, the chow was stored at -20°C until use. Fresh food from the freezer was provided to the animals every day while food remaining from the previous day was removed. The appropriate quantity (corresponding to daily uptake) of this high cholesterol diet was first weighed, mixed thoroughly with the appropriate amount of sesame oil and then dried and weighed again in order to achieve a final concentration of sesame oil in mice diet at a level of 10%.

Mice had free access to eat and drink throughout the study. During the 8-week period, food consumption was monitored daily, whereas the amount of water consumed daily was also recorded in HCN group. Mice were weighed once a week.

### Blood collection and serum lipid measurement

Blood samples of mice were collected at the beginning and at the end of the experimental period (at 9:00 am, after a 12-h fasting period), using capillary tubes introduced into the medial retro-orbital venous plexus under light ether anaesthesia. A quantity of about 150-200 μl of blood was collected from each mice at the beginning of the experiment. Bigger quantity of blood (400 - 500 μl) was collected at the end of the experiment and before mice's euthanasia. Serum was separated by centrifugation at 3000 rpm for 10 min and was stored at -80°C until analysis.

Serum concentrations of total cholesterol and of triglycerides were determined using the enzymatic PAP commercial kit ("biosis" - Biotechnological Applications, Athens, GR) and HDL-cholesterol was determined with a cholesterol enzymatic photometric method. LDL-cholesterol was determined by the mathematic model "LDL-cholesterol = Total Cholesterol- (HDL-cholesterol + Triglycerides/5)".

All samples were analyzed at the Laboratory of Experimental Surgery and Research of the Medical School of Athens (Athens, Greece).

### Serum lipid peroxidation - NO levels determination

Total peroxide concentration in collected serum was assessed photometrically (PerOx kit, Immundiagnostic AG, Bensheim, Germany). Total serum nitric oxide (NO) was calculated based on the enzymatic conversion of nitrate to nitrite by nitrate reductase, using a commercial kit (Total Nitric Oxide and Nitrate/Nitrite Parameter Assay Kit, R&D SYSTEMS, Minneapolis, MN, U.S.).

### Histopathological staining

At the end of the 8-week period, animals were euthanized under ether anaesthesia with their livers and aortas dissected immediately for further histopathological analysis. Part of the livers and aortas were fixed in 10% formalin at room temperature. The tissues were then embedded in paraffin, sectioned and mounted on glass microscope slides. The sections were stained with hematoxylin-eosin and examined blindly by two independent researchers using light microscopy. More precisely, the liver was evaluated as had been previously described by the Pathology Committee of non-alcoholic steatohepatitis Clinical Research Network [8]. The histological features were grouped into 4 broad categories: steatosis, ballooning, portal inflammation and lobular activity. A score from 0 (absence) to 3 (severe lesion) was assigned to each parameter.

### Statistical evaluation

All of the analyses were performed using the SPSS statistical software. The results were expressed as mean values ± standard errors and analyzed for statistical significance by One Way ANOVA test. Liver lesions were demonstrated as mean score ± standard errors for steatosis, ballooning, portal inflammation and lobular activity. Statistical significance was set at *P *< 0.05.

## Results

### Body weight and food and water intake

In the present study the initial body weight for all groups was similar. At the end of the 8-week study the mice of the HC group presented reduced body weight compared to the animals in the NC (*P *= 0.027), HCN (*P = 0.018*) and HCS (*P = 0.002*) groups (Table 1). A statistically significant increase was observed in food consumption in the HCN group relative to the NC (*P *< 0.001) and HCS (*P *< 0.001) group. The daily water volume consumed by the mice in HCN group was 4.36 ± 0.15 mL.

**Table 1:** Body weight and food consumption

		Groups			
Measurements		NC	HC	HCN	HCS
Initial Body Weight (g)		23 ± 0.45	22.40 ± 0.40	22.85 ± 0.40	23.50 ± 0.33
Final Body Weight (g)		24.3 ± 0.61^b^	22 ± 0.63^acd^	24.28 ± 0.52^b^	25.25 ± 0.53^b^
Food Consumption (g/day)		3.25 ± 0.03^a^	3.38 ± 0. 04	3.52 ± 0. 05^ad^	3.23 ± 0. 05^a^
Total cholesterol (mg/dL)	Time 0	94.00 ± 6.31	85.50 ± 7.34	83.25 ± 4.33	88.62 ± 5.22
	Time 1	95.60 ± 4.15^bcd^	203.4 ± 11.29^ac^	158.57 ± 5.38^ab^	188.12 ± 15.09^a^
LDL-cholesterol (mg/dL)	Time 0	22.13 ± 3.83^bc^	8.00 ± 3.61^a^	9.85 ± 2.09^a^	16.87 ± 1.65
	Time 1	23.72 ± 3.93^bcd^	147.20 ± 9.95^ac^	107.62 ± 4.54^abd^	140.72 ± 13.07^ac^
HDL-cholesterol (mg/dL)	Time 0	48.83 ± 3.80	53.00 ± 5.56	50.25 ± 1.95	50.50 ± 2.22
	Time 1	56.20 ± 1.59^d^	49.20 ± 4.60	44.28 ± 1.65	41.75 ± 3.85^a^
Triglycerides (mg/dL)	Time 0	115.16 ± 9.86	139.50 ± 15.84	121.50 ± 8.57	137.25 ± 7.86
	Time 1	78.40 ± 8.58^bcd^	35.00 ± 1.81^a^	33.28 ± 1.70^a^	28.25 ± 1.58^a^

Body weight at the beginning and at the end of the experimental study, food consumption levels and total cholesterol, LDL-cholesterol, HDL-cholesterol and triglycerides levels in serum before (Time 0) and after the 8-week experimental period (Time 1)

(NC), Control mice; (HC) mice fed the high cholesterol diet; (HCN) mice fed the high cholesterol diet and treated with NAC; (HCS) fed the high cholesterol diet enriched with sesame oil.

a: Significantly different from NC group

b: Significantly different from HC group

c: Significantly different from HCN group

d: Significantly different from HCS group

Values are presented as means ± standard error, *P *< 0.05.

### Serum lipid profile

At the initial blood sampling, no differences in lipid levels (except LDL-cholesterol, Table 1) were recorded among the groups. Feeding of the high cholesterol diet containing 2% cholesterol and 0.5% cholic acid for the period of 8 weeks resulted in the development of hyperlipidemia in experimental mice groups compared to the control group. Serum total cholesterol and LDL-cholesterol levels (Table 1) were significantly increased in the three groups fed the high cholesterol diet compared to the NC group (*P *< 0.001).

NAC co-administration markedly reduced total cholesterol (*P *= 0.013) and LDL-cholesterol (*P *= 0.012) levels compared to the HC group. Although there was a slight decrease in total cholesterol and LDL-cholesterol in HCS compared to the HC group, this reduction was not statistically significant. HDL-cholesterol levels were found to be decreased in HCS group compared to NC group (*P *= 0.006), whereas there was no difference between the other groups. Lower concentrations of triglyceride levels were observed in all groups fed the high cholesterol diet in comparison to the control group (*P *< 0.001).

### Serum lipid peroxidation - NO levels determination

**Figure 1:**
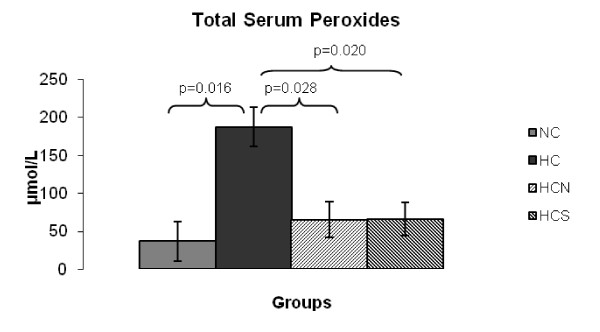
**Total serum Peroxides (μmol/L) at the end of the 8-week experimental period**. (NC), Control mice; (HC) mice fed the high cholesterol diet; (HCN) mice fed the high cholesterol diet and treated with NAC; (HCS) fed the high cholesterol diet enriched with sesame oil, *P *< 0.05.

**Figure 2:**
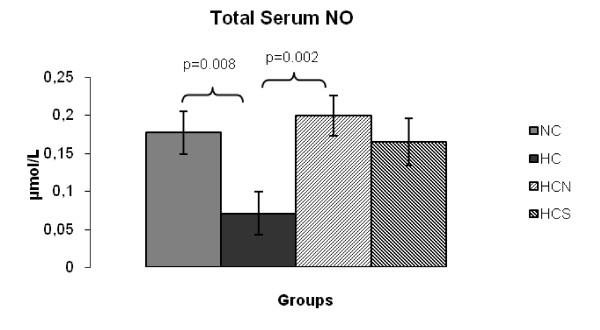
**Total serum NO (μmol/L) at the end of the 8-week experimental period**. (NC), Control mice; (HC) mice fed the high cholesterol diet; (HCN) mice fed the high cholesterol diet and treated with NAC; (HCS) fed the high cholesterol diet enriched with sesame oil, *P *< 0.05.

The evaluation of peroxidation products showed that peroxide content in the serum was markedly altered in the HC group related to the other three experimental groups (Figure 1). Both NAC and sesame oil administration reduced serum peroxidation to the levels of the control group, improving antioxidant status (Figure 1). NO serum levels (Figure 2) were reduced in the HC group in comparison to the control group, whereas the difference was marginal between the HC and the HCS group. Nevertheless, the HCN group presented significantly elevated NO levels compared to the HC group preserving it at its normal level for mice.

### Liver and aorta histopathology

**Figure 3:**
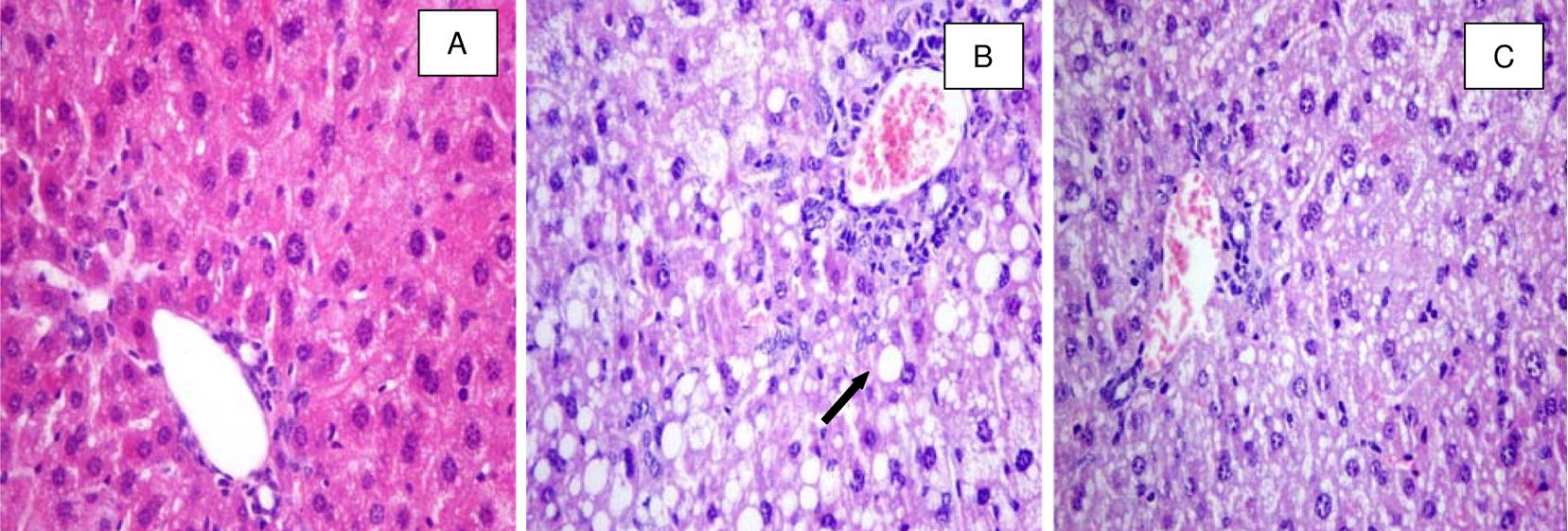
**Haematoxylin and eosin staining of hepatic tissue (400×)**. Control mice (A); mice fed high cholesterol diet (B); mice fed the high cholesterol diet and treated with NAC (C). Arrow in B. indicates hepatocyte lipid inclusion (steatosis). Note the dark periportal inflammatory infiltrates in B. and C.

Hematoxylin-eosin stained liver samples, obtained from the groups fed the high cholesterol diet, showed signs of liver steatosis and inflammation (Figure 3). A significant increase in accumulation of fat droplets was observed in the livers of the HC and HCS groups compared to the NC group [HC and HCS groups vs NC, (*P *= 0.001) and (*P *< 0.001) respectively] (Table 2). This increase was apparently suppressed in HCN group of mice in comparison to the HC group (*P *= 0.001), whereas no difference was observed between HCN and NC groups (*P *= 0.763). Moreover, the architecture of hepatocytes in the HCN group was found to be more similar to NC group compared to that of the HC and HCS groups (Figure 3). Ballooning of hepatocytes and lobular activity were observed in liver tissues of all mice in the HC and HCS groups, as compared to only two of the seven animals of HCN group. Neither NAC nor sesame oil administration improved hepatic inflammation. The grade of fatty liver disease was considered as "mild" in three of the seven mice in HCN group. In five mice from the HC group, the grade was "severe" in three, "moderate" in one and "mild" also in one mouse.

**Table 2:** Histological observations of hepatic tissue

Groups	Steatosis	Ballooning	Portal inflammation	Lobular activity	Overall
NC	0.00 ± 0.00^b,d^	0.00 ± 0.00^b,d^	0.00 ± 0.00^b,c,d^	0.00 ± 0.00^b,d^	0.00 ± 0.00^b,c,d^
HC	2.00 ± 0.31^a,c,d^	1.40 ± 0.40^a,c,d^	1.80 ± 0.58^a^	1.20 ± 0.20^a,c^	6.40 ± 0.87^a,c,d^
HCN	0.28 ± 0.18^b,d^	0.28 ± 0.18^b,d^	1.28 ± 0.47^a^	0.28 ± 0.18^b,d^	2.14 ± 0.59^a,b,d^
HCS	3.00 ± 0.00^a,b,c^	2.12 ± 0.29^a,c^	2.00 ± 0.00^a^	1.87 ± 0.29^a,c^	9.00 ± 0.18^a,b,c^

3, severe; 2, moderate; 1, mild; 0, no changes in histology (scores)

(NC), Control mice; (HC) mice fed high cholesterol diet; (HCN) mice fed the high cholesterol diet and treated with NAC; (HCS) fed the high cholesterol diet enriched with sesame oil.

a: Significantly different from NC group

b: Significantly different from HC group

c: Significantly different from HCN group

d: Significantly different from HCS group

Values are presented as mean score ± standard error, *P *< 0.05.

In evaluating the total examined parameters in hepatic tissue of mice, we conclude that NAC administration improved liver histology whereas diet supplementation with sesame oil had negative effects.

No fatty streaks or plaques were recorded in aortic tissues of all experimental mice (data non shown).

## Discussion

Hypercholesterolemia is one major risk factor for the development of atherosclerosis. The present study focused on the possible antilipidemic effect of NAC and sesame oil administration in hypercholesterolemic mice. To the best of our knowledge, this is the first study that investigated NAC and sesame oil action on the lipid profiles of mice consuming a chow diet enriched with cholesterol and cholic acid.

Cholesterol-cholic acid feeding has often been used to raise cholesterol levels in plasma and tissues of experimental animals [9,10]. The experimental diet followed in the present study resulted in elevated total cholesterol and LDL-cholesterol levels in all mice receiving it. Triglyceride levels decreased in all groups fed the high cholesterol-cholic acid diet. This finding is in accordance with previous studies in mice and rat groups fed with diets containing cholic acid or cholate [11,12]. Furthermore, according to the study of Kamisako et al. [13] there was a slight decrease in triglyceride levels of rats fed a diet containing 1% cholic acid as compared to their control counterparts. Li et al. [11] assumed that the triglycerides reduction in their study might be a consequence of the inhibition on 7a-hydroxylase activity due to the presence of the cholate in the experimental atherogenic diet, something that is not in accordance with Beigneux et al. [14] who suggested that inhibition of 7a-hydroxylase activity leads to the increase of triglyceride levels.

Hypercholesterolemia enhances the free radical generation in various ways [1] and is generally associated with an increased production of oxygen radicals such us superoxide anion radical [15]. NAC in our study had beneficial effects concerning lipid profile. The administration of this antioxidant largely prevented the diet induced increase in serum lipids (total cholesterol and LDL-cholesterol levels).

NAC has been shown to reduce cholesterol levels in plasma and liver in Balb/cA mice consuming a high saturated fat diet [2]. Krieger et al. [16] mentioned a slight reduction in plasma lipid fractions by means of NAC supplementation in hypercholesterolemic LDL receptor knockout mice. Therefore, other researchers indicate a beneficial effect of NAC on dyslipidemic profile of rats fed a high sucrose diet or of rats given standard chow and 30% sucrose in their drinking water [17,18]. Putative mechanisms accounting for the lipid-lowering effects of NAC might be related to its antioxidant properties. NAC in our study led to reduced serum peroxide content. The maintenance of the normal structure of lipoprotein receptors is indispensable for their function, improving the cellular uptake of serum lipids from the blood. Reactive oxygen species produced during oxidative stress react with lipoproteins to produce oxidation states, diminishing the cellular uptake of lipids from the blood [17,19,20]. Thus the antioxidant action of NAC might contribute to elevated cellular lipid uptake, resulting in the decrease of serum cholesterol levels.

According to Lin and Yin [21], the lipid lowering action of NAC in mice consuming a high fat diet is attributed partially to the suppression of mRNA expression of three lipogenic-related enzymes (malic enzyme, fatty acid synthase and 3-hydroxy-3-methylglutaryl coenzyme A reductase).

NO is considered as an important vasoactive substance which protects the integrity of the blood vessels. Hu et al. [22] concluded that plasma NO levels declined in rabbits fed a high fat diet. Similar to this study, we observed that our experimental high cholesterol-cholic acid diet suppressed serum NO levels, whereas NAC administration raised NO bioavailability and maintained it at the normal level. Thus NAC might display an essential protecting role for the normal function of arteries.

Our results concerning hepatic steatosis and inflammation are consistent with those of previous investigations [23]. As previously described, NAC administration suppresses hepatic lipid infiltration in hepatic tissues of mice fed high fat diets [21]. The hepatoprotective action of NAC might be associated with its antioxidant action, as mentioned above.

Similar to the findings of Sener et al. [9], the cholesterol-cholic acid diet used in our study did not lead to fatty streak or plaque formation in aortic tissues of mice. This finding was partially anticipated, as our test diet consisted of the regular chow diet (3.5% fat) enriched with cholesterol and cholic acid without additional fat. In contrast, diets used for the initiation of atherosclerosis in wild-type mice like Paigen diet [24,25] have a final fat composition of more than 15% (in weight percentage).

Sesame oil supplementation, at a level of 10% in the test diet, failed to restore the disturbed lipid profile caused by the high cholesterol-cholic acid diet, despite the decrease in serum peroxidation. Total cholesterol and LDL-cholesterol levels exhibited a decrease by sesame oil administration. This preventive effect however could not be statistically confirmed, probably due to the limited number of available observations or/and to the elevated caloric value of the sesame oil modified diet. In addition, we observed that sesame oil co-administration enhanced hepatic steatosis and inflammation.

Administration of dietary defatted sesame flour to rabbits did not protect against cholesterol-induced hypercholesterolemia, but decreased the susceptibility to oxidative stress (lower serum and liver lipid peroxidation) perhaps due to the antioxidant activity of sesaminol [26]. Bhaskaran et al. [27] reported a significant reduction in plasma cholesterol, LDL-cholesterol and triglycerides levels in LDLR-/- mice when their atherogenic diet was reformulated with the same level of sesame oil. In our study, sesame oil was added to the test diet without fat substitution. The additional calories of the sesame oil diet might resulted in the absence of hypolipidemic response, as mentioned above.

In an earlier study, an increase was shown in the hyperlipidemic status of rabbits fed a hypercholesterolemic diet enriched with olive oil [28], whereas Acin et al. [29] concluded that dietary cholesterol suppresses the ability of olive oil to delay the development of atherosclerotic lesions in mice, negatively influencing their plasma lipid parameters. Therefore we can assume that cholesterol addition, like in the case of olive oil, in part suppresses the putative hypolipidemic action of sesame oil.

In summary, even though sesame oil presented antioxidant properties, it did not show significant hypolipidemic activity in mice consuming a chow diet enriched with cholesterol and cholic acid. NAC administration was able to improve lipidemic status in the serum, to maintain liver physiology and to reduce serum peroxides probably by ameliorating the efficiency of the antioxidant defence system of experimental animals. Moreover, NAC might exert preventive antiatherogenic activity by means of raised serum NO levels.

## Conclusions

Co-administration of NAC, but not sesame oil, restored the disturbed lipid profile and hepatic injury in the studied hypercholesterolemic murine model, although both agents enhanced serum antioxidant capacity. Thus NAC might represent a beneficial alternative for lowering cholesterol levels in humans during clinical practice.

## Abbreviations

(NAC): N-acetylcysteine; (NC): control group; (HC): group receiving test diet supplemented with cholesterol and cholic acid; (HCN): group receiving the test diet with NAC supplementation; (HCS): group fed the test diet enriched with sesame oil; (LDL): low density lipoprotein; (HDL): high density lipoprotein; (NO): nitric oxide.

## Competing interests

The authors declare that they have no competing interests.

## Authors' contributions

LMK has been involved in experimental design, animal experiments, laboratory analysis, data collection and statistical analysis. GA carried out the histopathological examination. AP has been involved in experimental design and animal experiments. ISV participated in experimental design and performed part of the statistics. DI and TK have been involved in experimental design. DNP participated in experimental design, animal experiments, laboratory analysis and data collection. All of the above helped in drafting the manuscript. All authors read and approved the final manuscript.
